# Denitrification Modularity and Its Environmental Controls in Transiently Versus Permanently Anoxic Marine Systems

**DOI:** 10.1111/1462-2920.70407

**Published:** 2026-08-21

**Authors:** J. Catherine Hexter, Weiyi Tang, Samantha G. Fortin, Amal Jayakumar, Bess B. Ward

**Affiliations:** ^1^ Department of Geosciences Princeton University Princeton New Jersey USA; ^2^ College of Marine Science University of South Florida St. Petersburg Florida USA

**Keywords:** anoxia, denitrification, metagenomic, modularity

## Abstract

Denitrification is a modular process that is mediated by an assemblage of microbes with varying denitrification gene combinations. The controls on these gene combinations, known as modularity, are poorly understood and marine observations are mostly limited to permanently anoxic systems. In this global metagenomic analysis representing 69 water column metagenome samples we report different modularity patterns associated with environmental parameters based on the permanence of anoxia. Thermodynamic favourability alone is not enough to explain the distribution of modularity patterns. Instead, variables such as the permanence (or transience) of anoxia, oxygen availability, biogeography and ratios of organic matter to nitrogen supply all help shape the denitrifier community gene assemblage. Environmental correlates in transiently anoxic compared to permanently anoxic systems suggest that the pressures of a more complex environment may favour shorter pathways due to resource allocation trade‐off regardless of organic matter availability. Nitrate reduction is the dominant step compared to the rest of the denitrification pathway irrespective of anoxia type. As increases in global temperature result in more seasonally anoxic and hypoxic waters, these results highlight the importance of understanding the controls on denitrification modularity under varying states of anoxia.

## Graphical abstract

Denitrification modularity is controlled by several environmental factors, especially concentrations of carbon and nitrogen substrates. In permanently anoxic environments, longer pathways are favored by high carbon/nitrate ratio, consistent with thermodynamic expectations and ecological models. In seasonal and transient anoxia, environmental complexity alters modularity patterns, possibly due to energetic tradeoffs.

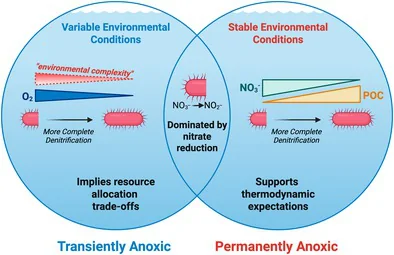

## 1 Introduction

In the predominantly nitrogen (N) limited open ocean, N loss processes such as denitrification and anammox control the fixed N inventory, which in turn modulates primary production. Denitrification and anammox are thermodynamically favourable in low oxygen environments, with ocean oxygen minimum zones (OMZs) estimated to be responsible for up to 50% of marine fixed N loss (DeVries et al. 2013). Denitrification, specifically, has been found to be the dominant form of N loss at the majority of depths in the Arabian Sea (Ward et al. 2009), Eastern Tropical South Pacific (ETSP) and Eastern Tropical North Pacific (ETNP) OMZs (Dalsgaard et al. 2012; Babbin et al. 2014, 2020). In eutrophic hypoxic or anoxic coastal waters, denitrification can often be beneficial as it counteracts the deleterious effects of excess dissolved inorganic nitrogen (DIN) input (Zheng et al. 2021; Gao et al. 2022).

Denitrification is a step wise microbial respiratory pathway (NO_3_^−^ → NO_2_^−^ → NO → N_2_O → N_2_). These steps are each catalysed by individual reductase enzymes, nitrate reductase (Nar), nitrite reductase (Nir), nitric oxide reductase (Nor) and nitrous oxide reductase (N2OR), which are encoded by the functional genes *narG*/*napA*, *nirS*/*K*, *norB* and *nosZ*, respectively. Denitrification is modular, that is, different microbes perform different steps of the pathway in a synergistic fashion, and partial denitrifiers outnumber complete denitrifiers (Graf et al. 2014; Roco et al. 2017; Zhang et al. 2023; Pold et al. 2024). The modularity (variable combinations of denitrification genes) of this pathway has important implications for N loss or retention (e.g., reducing NO_3_^−^ only as far as NO_2_^−^ retains fixed nitrogen), as well as emissions of the potent greenhouse gas and ozone‐depleting substance, nitrous oxide (N_2_O). Additionally, individual intermediate steps encoded by the genes *nir* and *nor* can be involved in detoxification of toxic intermediates NO_2_^−^ and NO, respectively, and all denitrification genes may support continued election transfer by consuming electrons from reduced cytochromes, thus allowing for their subsequent reoxidation (Chen and Strous 2013; Vázquez‐Torres and Bäumler 2016). The distribution of denitrification gene patterns (modularity) is not random; both community structure and habitat can be predictive of modularity, and in turn, may be predictive of potential N loss and N_2_O fluxes in terrestrial ecosystems (Graf et al. 2014). However, denitrification modularity and its controls are still poorly understood and sparsely explored in aquatic ecosystems.

Many factors have been proposed to influence denitrification modularity in the environment. The dominant hypothesised controls on modularity include: the available thermodynamic energy (Δ*G*) of each nitrogen species intermediate; the amount of that available energy that can be captured based on the electron transport chain (ETC) configuration and cost of energy production (H+ pumped per 2e^−^ donated); the relative availability of electron donor (organic matter; OM) versus electron acceptor (DIN); and the energetic and metabolic versatility trade‐offs of maintaining a long ETC (Chen and Strous 2013; Simon and Klotz 2013; Sun et al. 2024; Roothans et al. 2025).

While the denitrification intermediate with the greatest Δ*G* is N_2_O, only nitrate reductase, encoded by *narG*, can directly contribute to a proton motive force, as the other enzymes in the chain require cytochromes and quinols for proton transport (Simon et al. 2008). While both NarG and NapA can pump the same number of protons, NapA relies on a quinol pool and often has lower energy yield but is more suited for low nitrate environments given its scavenging capabilities (Potter et al. 1999; González et al. 2006). While the exact bioenergetics of Nar and Nap rely on their accessory genes, this first step has the highest potential for thermodynamic favourability and relies on a stable, often abundant, non‐toxic substrate (González et al. 2006). For this reason, it is hypothesised that NO_3_^−^ reduction is the most thermodynamically favourable single step pathway. For complete denitrifiers, the reduction of 2 NO_3_^−^ all the way to N_2_ can translocate up to 30 protons but requires electron donation at each step (10e^−^) (Chen and Strous 2013). Therefore, in a carbon limited environment the amount of electrons that can be contributed to the respiratory ETC controls ATP yield, and as such, the energy yield for an organism with the complete denitrification pathway is the same as the yield in an organism with only one step (6H+/2e^−^) (Simon and Klotz 2013; Roothans et al. 2025). Therefore, in environments where electron acceptors (DIN) are in excess of electron donors (OM), shorter pathways are favoured and, inversely, complete denitrification is favoured in DIN limited and OM enriched environments. Metagenomic findings of highly abundant truncated denitrification pathways in OMZs and across global biomes suggest an environmental selection for shorter ETCs, but the environmental drivers of this modularity have been difficult to resolve (Zhang et al. 2023; Pold et al. 2025). A recent ecological model of OMZs has further supported these thermodynamic expectations, demonstrating changes in modularity niches along an OM:NO_3_^−^ gradient, with shorter pathways favouring high DIN and longer pathways favouring high OM (Sun et al. 2024). However, both culturing and metagenomic attempts to directly connect OM:NO_3_^−^ ratios to denitrification modularity have led to inconsistent results (Pessi et al. 2022; Roothans et al. 2024; Pold et al. 2025).

The environmental controls on modularity remain elusive and cannot easily be explained by resource availability and thermodynamic favourability alone. A more recent hypothesis suggests that resource allocation tradeoffs may be responsible for favouring partial over complete denitrification in unstable environments (Roothans et al. 2025). The importance of such tradeoffs is based on accumulating evidence that slower growing microbes, like those in natural assemblages, favour putting resources towards flexibility of carbon metabolism, antibiotic tolerance, uptake of micronutrients and stress response mechanisms over more expansive and complete metabolisms such as complete denitrification (Andersson and Levin 1999; DeBruijn 2016; Roop et al. 2016; Kreft et al. 2020). In stable high OM environments, such as laboratory cultures, complete denitrification is favoured because there is no risk or metabolic tradeoff involved in putting all resources towards energy generation. By this metric, one would hypothesise that irrespective of OM or DIN availability, high flux and variable/complex environments should favour shorter pathways while stable environments favour more complete denitrification.

A recent metagenomic study investigated modularity in the relatively stable environment of the permanently stratified anoxic zones of OMZs (Zhang et al. 2023). In comparison, many estuarine and coastal water systems experience seasonal and transient anoxia that can support denitrification (Hietanen and Kuparinen 2008; Walker et al. 2010; Howarth et al. 2011; Ji, Frey, et al. 2018; Tang, Fortin, et al. 2025). This transient anoxia is often brought on by increased stratification due to summer heating, as well as increased OM decomposition (Diaz and Rosenberg 2008). The transience of anoxia is often correlated with a variable flux of OM and inorganic nutrients (Gooday et al. 2009; Dai et al. 2023), making the transiently anoxic sites more variable/complex environments compared to permanently anoxic sites. The modularity of denitrification in these transiently anoxic aquatic environments remains largely unknown.

The degree and patterns of denitrification modularity are hypothesised to depend on factors such as OM, DIN and the stability of an environment (Ji, Frey, et al. 2018; Kreft et al. 2020; Roothans et al. 2025). We hypothesise that transient anoxia will alter the modularity of the denitrification community by providing an additional environmental pressure on metabolic flexibility that is not present in permanently anoxic systems such as OMZs (where investigation of modularity in the marine environment has been previously focused). To test his hypothesis, we assembled 2303 metagenomically assembled genomes (MAGs) from both stable aquatic environments that are permanently stratified and anoxic (i.e., OMZs) and from highly variable aquatic environments that are seasonally anoxic (e.g., Chesapeake Bay) in order to compare the denitrification modularity patterns between the two environment types. We also evaluated the influence of OM supply and DIN availability on modularity in both permanent and transient anoxia. Transiently anoxic systems are both high in OM:NO_3_^−^ ratios, which would favour long pathways based on thermodynamic expectations, and low in environmental stability, which would favour short pathways based on the resource allocation trade‐off hypothesis. Therefore, comparing them to low OM and more stable OMZs can help elucidate the controls on denitrification modularity.

## 2 Materials and Methods

### 2.1 Sample Selection and Sequencing

Metagenomic sequences of particulate material (> 0.22 μm) from the water column of sites with contrasting characteristics were selected to test the hypotheses about denitrification modularity under differing systems of anoxia. All samples were collected using standard oceanographic techniques from a rosette system equipped with Niskin bottles and a CTD profiler to record parameters such as temperature, salinity, pressure and in situ oxygen concentration. All water samples were filtered onto 0.22 μm filters and frozen for downstream DNA analysis. Samples were selected to represent permanently anoxic (mainly open ocean) as well as transiently anoxic (mainly estuarine) systems. Three depth features were sampled from each location, representing (1) the oxic surface layer, (2) the bottom of the oxycline and (3) the core of the oxygen depleted layer. The oxic surface layer was defined as depths within the fully oxygenated mixed layer. The oxycline samples were defined as at the inflection point of rapid oxygen decrease in the oxygen concentration profile (all samples were collected at oxygen concentrations below 85μM oxygen). The anoxic samples were defined as depths within the depth range at which oxygen concentration was below detection on the Seabird CTD probe. In depth profiles from transiently anoxic sites during the time of year when the water column was well mixed and fully oxic, depths were chosen based on the location of surface, oxycline and anoxic conditions during the anoxic period. Locations of sample sites are shown in Figure S1 and each metagenome sample and its associated metadata is listed in Table S1.

### 2.2 Permanently Anoxic Environments

Previously published metagenomes were reanalysed from three major OMZs, the Eastern Tropical North Pacific (ETNP), the Eastern Tropical South Pacific (ETSP) and the Arabian Sea (AS), along with the Cariaco Basin and the Black Sea. Four depth profiles were selected from the ETNP: two profiles of stations PS2 and PS3 from the SR1805 Cruise in April of 2018 (Fortin et al. 2024), one profile from the R/V New Horizon cruise in June of 2013 (NH‐1315) (Glass et al. 2015), and one profile from the Oxygen Minimum Zone Microbial Biogeochemistry Expedition (OMZoMBiE) cruise (R/V Horizon, 13–28 June 2013) (Tsementzi et al. 2016). A complete profile (containing surface, oxycline and anoxic samples) was not available from the OMZoMBiE cruise, so only two depths (oxycline and anoxic) were included. The only depth profile deeply sequenced enough for high‐quality metagenomic assembly from the ETSP was collected in July 2013 on the R/V Nathaniel Palmer 1305 (Sun and Ward 2021). One depth profile from Station 2 on the 2007 KNOX009 cruise to the Arabian Sea was also included, and notably is missing both an oxygenated surface sample and oxycline sample, as all four samples were collected below the oxycline (Fortin et al. 2024). Samples were also included from two depth profiles in the Cariaco Basin collected during May and November of 2016 (Geller‐McGrath et al. 2023) and one Black Sea depth profile from station 307 collected in October 2019 (Cabello‐Yeves et al. 2021).

### 2.3 Transiently Anoxic Environments

Previously reported metagenomic data were reanalysed from Chesapeake Bay (CB) from August 2020 at station CB1.5, representing a seasonally anoxic and stratified depth profile (Tang, Hexter, et al. 2025). New metagenomic data first reported here from station CB2 in October 2019 represented an unstratified fully oxic station at a nearby location. Samples were collected on the R/V Hugh Sharp on cruises HRS2002 and HRS1914, respectively. CB2 is a good control comparison because it was not anoxic at the time of sampling, but during the summer months experiences anoxia at depth, similar to the conditions observed at CB1.5 (Tang, Tracey, et al. 2022).

Previously published samples from Saanich Inlet (SI) in August of 2010 and January of 2011 (cruises 48 and 52, respectively) (Lin et al. 2021), and two stations from the July 2013 nGOM cruise (R/V Pelican) in the Gulf of Mexico (GOM) also represent transiently anoxic sites (Gillies et al. 2015).

Sampling and sequencing methods for all samples other than Chesapeake Bay CB2 samples have been previously described in the citations above. Metagenomic samples from CB2 are first reported here. DNA sample collection, metagenomic library prep and sequencing methods for CB2 were the same as described previously for CB1.5 (Tang, Jayakumar, et al. 2022). All Chesapeake Bay metagenomes used in this study have been deposited in NCBI GenBank under accession number PRJNA1151642. All newly assembled metagenomically assembled genomes (MAGs) in this study have been deposited to NCBI GenBank under BioProject number PRJNA1472692. Accession numbers, citations and metadata for all samples and MAGs, both new and previously published, are provided in Tables S1 and S2.

### 2.4 Metagenomic Assembled Genome Reconstruction

All metagenomically assembled genomes (MAGs) were reconstructed from reads except for the Cariaco Basin, in which previously assembled and binned MAGs that met the quality cut‐offs of this study (greater than 80% complete and less than 10% contaminated) were used due to the similarities in assembly and binning pipelines (Geller‐McGrath et al. 2023). The remaining metagenomes were reconstructed from reads that were quality filtered with default settings of illumineUtils. Samples with over a 90% pass rate were included in the assembly (Eren et al. 2013). MEGAHIT v.1.2.9 (Li et al. 2015) was then used to assemble reads from each sample individually because it resulted in the highest quality of preliminary assemblies compared to other available assemblers. The ETSP and GOM samples were co‐assembled due to low sequencing depth. Contigs for each assembly were binned using METABAT2, CONCOCT and Maxbin2 (Alneberg et al. 2014; Wu et al. 2016; Kang et al. 2019) via the metaWRAP pipeline v.1.3.2 (Uritskiy et al. 2018). The best binning algorithm was then independently chosen for each bin, based on contamination and completeness scores and dereplicated using metaWRAP refine. A dereplication cutoff of 98% was chosen to avoid inflation errors in read mapping while also retaining potential intraspecies functional gene diversity (Sieber et al. 2018). Bins were dereplicated with dRep v.3.4.3 (Olm et al. 2017). Taxonomy was assigned using the GTDB‐Tk pipeline v.2.4.0 (Chaumeil et al. 2022). Metagenome assembled genomes (MAGs) were quality checked using CheckM v.1.2.2 (Parks et al. 2015) and grouped into high, medium and low quality groups based on previously described cutoffs (The Genome Standards Consortium et al. 2017). Only MAGs with high‐med completeness scores (greater than 80% complete and less than 10% contaminated) were used for downstream analysis.

### 2.5 Functional Gene Annotation and Relative Abundance

MAGs were annotated for the denitrification genes *narG*, *napA, nirK*, *nirS*, *nor* (including *bnor*, *cnor*, *snor*, *nnor*, *gnor*, *enor* and *qnor* variants) and *nosZ* (Clades I, II and III) using previously curated HMMER files (Murali et al. 2021; Zhang et al. 2023; Intrator et al. 2024; He et al. 2025). Genes were annotated using anvi‐run‐hmms in anvi'o v.7.1 (Eren et al., 2020). While these HMMs were based on translated amino acid sequences using default parameters in anvi‐run‐hmms, discussion of subsequent gene presence and absence will refer to the gene, rather than the protein, because no proteomic data were used in this study.

Relative abundances of MAGs were calculated by mapping reads to the metagenomes with bowtie2 v.2.5.1 using sensitive ‘end—to—end’ parameters (Langmead and Salzberg 2012). The number of reads mapped to each sample was divided by the total number of reads in the sample as a proxy for abundance normalised to sequencing depth. Relative abundance was calculated as a function of both taxonomy and denitrification gene co‐occurrence patterns (i.e., the relative abundance of each MAG was grouped by co‐occurrence pattern and summed).

### 2.6 Denitrification Completeness Index (DCI)

A denitrification completeness index was calculated to quantify the degree of modularity for each sample. The index was calculated as follows:

$$\mathrm{DCI}=\frac{\sum \left(a_{i}*L_{i}\right)}{L_{\max}}$$

where *a* is the percent relative abundance (relative to all denitrification MAGs within a sample) of a given pattern *i*, *L* is the length of that pattern (from 1 to 4) and *L*_max_ is the maximum length possible, in this case 4. The higher the DCI, the more complete the pathway. Importantly, this metric is agnostic to which step is present and only provides information on the length of the pathway.

### 2.7 Phylogenetic Tree Reconstruction

All alignments and trees unless otherwise stated were created as follows. All genes were converted to amino acid sequences using default parameters in prodigal v.2.6.3 (Hyatt et al. 2010). Those amino acid sequences were then aligned using MAFFT v.7.525 with 1000 iterations and local pair settings (Katoh and Standley 2013). Maximum likelihood trees were then constructed in IQ‐TREE v.2.3.6 using the automated model selection mode ‘test’ with bootstrap setting 1000 (Kalyaanamoorthy et al. 2017; Minh et al. 2020). ModelFinder selected the following models for each tree: WAG+I + G4 for NirK; LG + F + I + G4 for NirS, bNor, cNor, eNor, gNor, qNor, sNor, N2OR, NarG/NapA/NxrB; and cpREV+F + I + G4 for nNor. Trees were visualised in iTOL v.7.4 (Letunic and Bork 2024).

### 2.8 Quality Control (QC) and Taxonomic Filtering

Any MAGs assigned to known nitrifiers or anammox bacteria were removed from further consideration due to their unlikely role in denitrification. All of these MAGs only contained *nirK*, whose function is unknown in nitrifiers (Casciotti and Ward 2001; Lund et al. 2012) or is used in the anammox pathway rather than denitrification.

Candidate gene hits identified from HMMs were further quality filtered using a variety of techniques depending on the gene.

### 2.9 Gene QC for *narG/napA*

Due to the high sequence similarity of *narG* and *napA*, HMMs for the individual genes often resulted in redundant hits. It was not possible to separate *narG* and *napA* based on phylogenetic similarity as was done for *nxr* and other DMSO genes, due to a lack of phylogenetic separation (e.g., some subclades of *nar* are closer to subclades of *nap* than to more phylogenetically distinct subclades of *nar*) and the remanent *napA* motif ‘TAT’ occasionally occurring in verified *narG* sequences (Ize et al. 2009; Li and Turner 2009). Since both NarG and NapA use the same substrate, produce the same product and are capable of generating a proton motive force, considering their genes together is functionally consistent and does not prevent the interpretation of their ecological role. Sequence similarity to reference sequences of NxrB (Fortin et al. 2024) were used to filter out false hits. For the remaining 4864 copies of NapA and 3014 copies of NarG, BLASTp (v.2.16.0) searches were performed against the NCBI nr database with an e‐value cutoff of 1e‐5 and ‐max_hsps 1 settings (Altschul et al. 1990, 2005). The top three hits were indexed such that any hit to a known related DMSO gene was removed and only sequences that matched key terms such as ‘nitrate reductase’ or logical variations thereof were retained for downstream analysis. Additionally, amino acid sequences that matched phylogenies to known nitrifiers were assumed to be NxrB and removed. Following BLAST filtration, 3014 and 1324 sequences of NapA and NarG remained, respectively. Manual filtration was then performed via inspection of alignments for conserved domains and further manual parsing of BLAST results to ensure sequences aligned to high quality reference hits and not automatically assigned genes from HMM annotations, which would have inadvertently resulted in a circular quality control method. After manual filtration, 2878 *napA* and 1270 *narG* gene hits remained. Due to the high sequence number, it was not feasible to perform quality control via visualisation of the phylogenetic tree.

### 2.10 Gene QC for *nirK* / *nirS*

Verified reference translated sequences of NirK (Pold et al. 2024; Intrator and Ward 2025) and NirS (Pold et al. 2024) were used to reconstruct a phylogenetic tree (see methods above). The inclusion of out groups such as NirN and NirF was used to help detect false positives. BLASTp searches were performed on any sequences that did not cluster closely with known references or outgroups using the same settings as above and were individually inspected for sequence alignment. The final NirS and NirK sequences including a representative subset of the reference sequences used are shown in Figures S2 and S3, respectively. Citations and GCA numbers for this subset of reference sequences are provided in Table S3.

### 2.11 Gene QC for *qnor/enor/bnor/cnor/gnor/snor*

Individual phylogenetic trees were reconstructed for each clade of Nor with appropriate reference sequences (Murali et al. 2021). Sequences that did not cluster with known references were confirmed via BLASTp with the settings described above or removed if no match was found.

### 2.12 Gene QC for *nosZ*

Reference clade I and clade II N2OR translated amino acid sequences (Intrator et al. 2024) were clustered with HMM hits from this study using CDHIT v4.8.1 with an identity cut‐off of 0.98 and an alignment coverage cut‐off of 0.9, as opposed to tree reconstruction, because many of these reference sequences were from primer‐based studies and much shorter than a full length HMM hit, making alignment difficult (Fu et al. 2012). All sequences that clustered with a known reference were assigned the clade of that reference. The resulting verified sequences were placed into a phylogenetic tree using the parameters described below to ensure proper clade assignment. Clade III was identified using the methods described in (He et al. 2025) and the motif finding python script available on the supplemental GitHub. The sequences that contained the motif used to identify the novel clade III did not group separately in this study, so we used the original clade assignments for all downstream analysis. The clade III candidate sequences are shown in Figure S4. Final assigned clades for each MAG containing the *nosZ* gene are listed in Table S4.

### 2.13 Statistics

All visualisations were made in R version v.2.2.1 unless stated otherwise (R Core Team 2025). The station map visualisation for Figure S1 was created in MATLAB v.R2024a (The MathWorks Inc 2024). All available environmental data and relative abundance co‐occurrence patterns were analysed for significant correlations using a Spearman correlation test with Hmisc package (https://hbiostat.org/r/hmisc/) and corr package with use = ‘pairwise.complete.obs’ because some environmental variables were not available for the entire data set and set to NA (Simko 2021). To compute the ratio POC:NO_3_^−^, NO_3_^−^ concentrations of zero were changed to 0.00001 to avoid infinite values. Plots were also visualised with corrplot. A principal co‐ordinate analysis (CAP) was performed using phyloseq (McMurdie and Holmes 2013) to assess the dissimilarity of modularity patterns using Bray Curtis dissimilarity calculated with the Vegan package (Dixon 2003). Adonis2 from Vegan was used to run a permutational multivariate analysis (PERMANOVA) to assess the statistical significance between the dissimilarity matrix of co‐occurrence relative abundance and various environmental parameters such as depth, O_2_ concentration and site type. Relative abundance heatmaps were made with Pheatmap and hierarchical clustering was done by Euclidian distance metrics (Raivo 2025).

### 2.14 Environmental Variables

Environmental variables were obtained or estimated for the stations or depths where metagenomics samples were collected (Table S1). Temperature, salinity, oxygen and nutrient concentrations were directly extracted from the publications, and the methods followed in each publication are summarised in Table S5. For the Chesapeake Bay metagenomic samples first reported here, details of measured rates, biogeochemical variables (Tang, Jayakumar, et al. 2022), and nutrient data (Tang, Tracey, et al. 2022) were measured in triplicates and previously reported. Net primary production (NPP) was calculated based on the vertically generalised production model (VGPM) using MODIS satellite products (Behrenfeld and Falkowski 1997) (https://orca.science.oregonstate.edu/index.php). Export production (EP) was estimated by multiplying NPP by export ratio (Laws et al. 2011). EP at specific depths was estimated using the ‘Martin Curve’ using the global average *b* value of 0.858 (Martin et al. 1987; Lauderdale and Cael 2021). Particulate organic carbon (POC) concentrations were extracted from a global ocean model based on neural network (Sauzède et al. 2016) and from field observations where available (Luo et al. 2014; Kaiser et al. 2017; Tang, Tracey, et al. 2022).

## 3 Results and Discussion

The metagenomic samples represent a range of oxygen concentrations and stability from permanently anoxic OMZs to seasonally anoxic estuaries and seasonally hypoxic ocean water columns (Gulf of Mexico). The samples range from low to high latitudes covering three major ocean basins, three marginal seas and two estuarine systems (Figure S1). A total of 1418 dereplicated medium‐high quality (average 90.5% complete and 2.25% contaminated) MAGs were reconstructed from 69 metagenomic samples representing 8 locations (Table S2). Following taxonomic and gene QC filtration, 858 of these MAGs had one or more denitrification genes (Table S6).

### 3.1 Phylogenetic and Denitrification Functional Gene Diversity

**FIGURE 1:**
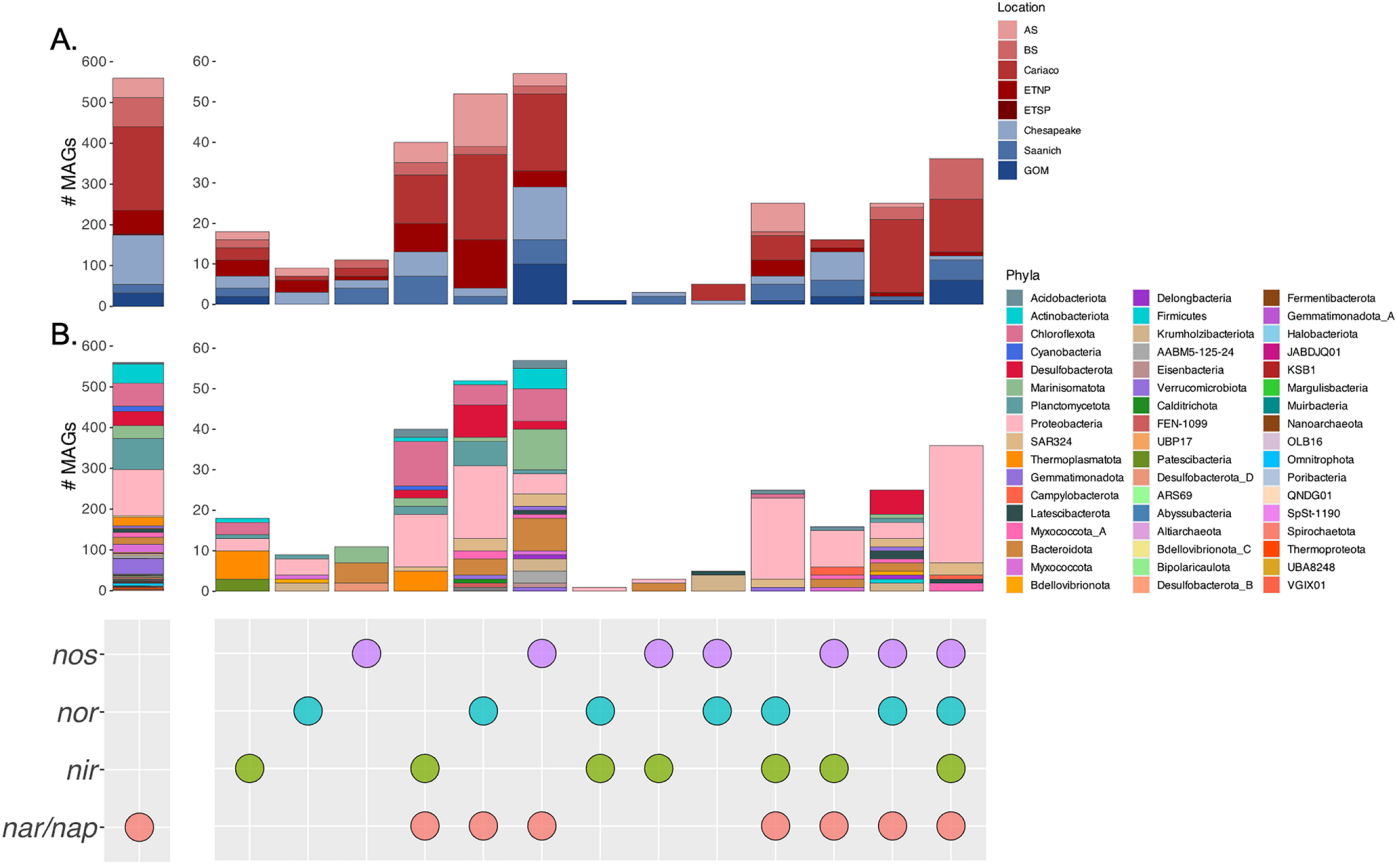
Total number of denitrification MAGs by sample location (A) and phylum (B). The bubble plot denotes denitrification gene presence to indicate the modularity type of each MAG, where each column is a distinct modularity pattern. ‘*nir*’ includes both *nirK* and *nirS*. Due to the high abundance of *nar/nap*, those MAGs are on a different scale than the remaining modularity types. Locations: Arabian Sea (AS), Black Sea (BS), Cariaco Basin (Cariaco), Eastern Tropical North Pacific (ETNP), Eastern Tropical South Pacific (ETSP), Chesapeake Bay (Chesapeake), Saanich Inlet (Saanich), Gulf of Mexico (GOM).

**FIGURE 2:**
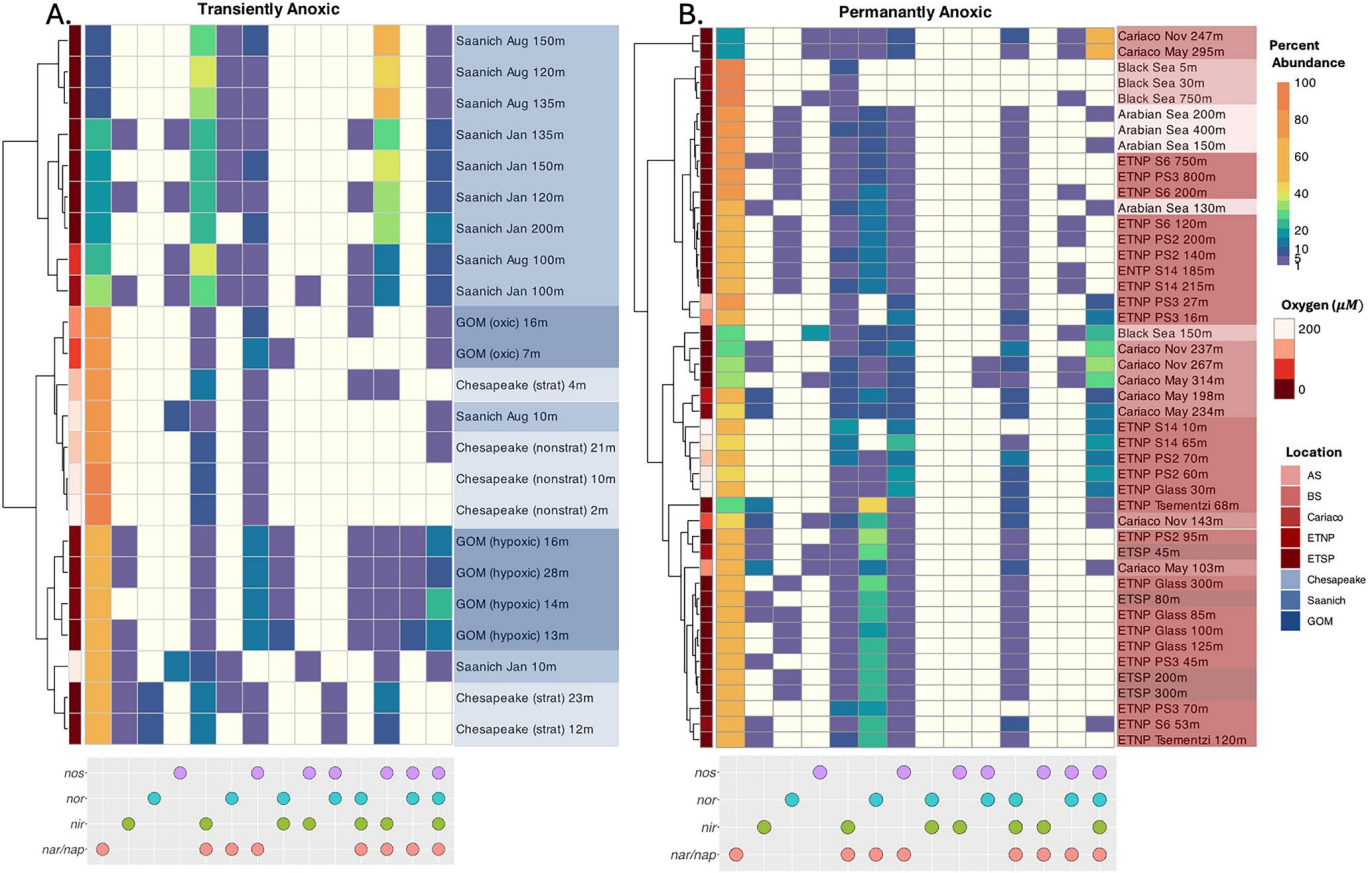
Heatmap of denitrifier relative abundance by modularity pattern. The relative abundance is calculated by modularity pattern abundance normalised to number of reads that mapped to MAGs with one or more denitrification genes for transiently anoxic (A) and permanently anoxic (B) samples. Modularity patterns less than 1% abundance are shown in white. The bubble plot denotes denitrification gene presence to indicate the modularity of each MAG. ‘*nir*’ includes both *nirK* and *nirS*. Oxygen concentration is shown in red and samples are hierarchically clustered by similarity of abundance patterns.

The most common modularity type was classified as *nar/nap*, with the majority of *nar/nap* MAGs reconstructed from Cariaco Basin and Chesapeake Bay (Figure 1a). Interestingly, despite the large number of *nar/nap* MAGs that were reconstructed from Cariaco Basin, the relative abundance of this modularity pattern, found by mapping all MAGs to the Cariaco metagenome, was the lowest of any permanently anoxic sample (Figure 2b). The *nar/nap* group was by far the most diverse modularity type with a Shannon diversity index of 2.18 compared to a mean diversity index of 1.41 (Table S7). The *nar/nap* MAGs represented 46 phyla but were dominantly *Proteobacteria*, *Chloroflexota*, *Actinobacteriota* and *Plantomycetota*. Importantly, while many bacteria in the *Plantomycetota* phyla are anammox bacteria, those known anammox organisms were removed from this study. The *Plantomycetota* MAGs visualised here were predominantly part of the *Phycisphaeraceae* and *Pirellulaceae* families which are not known to be anammox bacteria. The *nar/nap* MAGs and MAGs with *nar/nap* and only one other denitrification gene (i.e., *nar/nap_nir*, *nar/nap_nor* and *nar/nap_nos*), were phylogenetically distinct and diverse compared to other gene combinations (Figure 1b; Table S7). This suggests a distinct niche for nitrate reduction that is different from all other denitrification modularities, which has been previously reported in both metagenomic and rate measurements, where the nitrate reduction to nitrite rate greatly exceeds that of reduction to N_2_ (Tracey et al. 2022; Zhang et al. 2023).

The remaining three single gene pathways (*nir*, *nor* and *nos*) were far less prevalent than the *nar/nap* pathway (Figure 1). This result is in contrast to a previous modularity study, which found that all single gene pathways are very common, more so than any pathway with more than one gene (Zhang et al. 2023). However, this previous marine study used a much lower completion cutoff (50%), which could have resulted in an overrepresentation of truncated pathways.

The second most prevalent modularity type is *nar/nap_nos* which represents a striking amount of phylogenetic diversity and for which MAGs were reconstructed at all sites except the ETSP (Figure 1). A large portion of the *nar/nap_nos* MAGs are in the *Marinisomatota* phylum, which has been previously reported in an OMZ metagenomic study as containing many different denitrification modularities; however, none of the previously reported modularities included *nosZ* (Sun and Ward 2021). *Nar/nap_nos* MAGs were not found to be abundant in modularity studies that focus only on the three major permanently anoxic OMZs (Zhang et al. 2023) and cannot be quantified from a previous global modularity study because that study did not include *narG* or *napA* (Pold et al. 2025). Almost half of the *nar/nap_nos* MAGs included in this study were reconstructed from transiently anoxic sites (Figure 1). The permanently anoxic *nar/nap_nos* MAGs were predominantly reconstructed from Cariaco Basin. The two denitrification genes in this modularity are likely involved in a process known as ‘bet‐hedging’, in which one gene is turned on prior to anoxia in order to avoid entrapment in anoxia (Lycus et al. 2018). In a bet‐hedging scenario, microbes with these genes would be present and persistent in fully oxygenated environments that are turbulent or likely to become anoxic.

Complete denitrifiers and denitrifiers lacking *nosZ* (*nar/nap_nir_nor*) were two of the least phylogenetically diverse groups. We reconstructed only 37 MAGs capable of complete denitrification, comprising just over 4% of the total denitrifier MAGs, mostly reconstructed from the Cariaco Basin and the Black Sea (Figure 1). As the modularity increases in completeness, the diversity of the representative MAGs tends to decrease and become more dominated by *Proteobacteria* (Figure 1). *Proteobacteria* are a common group associated with complete denitrification (Graf et al. 2014; Zhang et al. 2023; Pold et al. 2025). These results provide further support for the growing body of evidence that complete denitrification is the exception and not the rule (Graf et al. 2014; Lycus et al. 2017; Roco et al. 2017; Pold et al. 2025; Roothans et al. 2025).

Interestingly, the *nir_nor_nos* modularity pattern is completely ‘missing’. A previous metagenomic study with lower cut‐offs was still only able to reconstruct one MAG with this modularity pattern (Zhang et al. 2023). Previous modelling work suggests that this pattern should fill a similar niche space as *nar/nap*, but is far more constrained by O_2_ and OM availability, which may explain why *nar/nap* is more dominant in the environment (Sun et al. 2024). Denitrification rates in incubation experiments using ^15^NO_2_^−^ to trace N_2_ production can be higher than those measured using ^15^NO_3_^−^ as the tracer (Tang et al. 2024) and most reported rates were measured using ^15^NO_2_^−^. High rates of denitrification from ^15^NO_2_^−^ led to the hypothesis that *nir_nor_nos* should be an important modularity pattern. In contrast, N_2_O production rates in incubation experiments using ^15^NO_3_^−^ to trace N_2_O production are often higher than those using ^15^NO_2_^−^ as the tracer (Ji et al. 2015; Ji, Frey, et al. 2018; Tang, Tracey, et al. 2022).

Modularity may explain these observations suggesting that rates of N_2_ and N_2_O production from ^15^NO_3_^−^ and ^15^NO_2_^−^ are not mediated by one individual organism but rather a suite of denitrifiers working together. This is compatible with the observation that the *nir_nor* type is also in very low abundance across all sites (Figure 2). Multiple independent modular steps involving cross feeding among microbes would potentially involve isotope dilution of the ambient intermediate DIN pools, depending on the size of those in situ pools and it is hard to evaluate the effect of such dilution on measured rates. An alternative explanation could be related to bias in MAG reconstruction because high strain level heterogeneity in denitrification modules may lead to fragmented assemblies and result in a bias towards reconstruction of partial denitrification over complete denitrifiers. More work is needed to understand why this modularity is missing from these systems.

### 3.2 Denitrification Modularity Patterns Differ With Stability of Anoxia and Stratification

The relative abundance of modularity patterns differs between permanently and transiently anoxic systems. The overrepresentation of *nar/nap* modularity is stronger in permanently anoxic sites than in transiently anoxic sites (Figure 2). Additionally, in the transiently anoxic sites, the lowest abundances of *nar/nap* cluster together and represent the anoxic samples while the fully oxic samples have the highest abundances of *nar/nap*. Conversely, in the permanently anoxic sites, the samples with the lowest abundances of *nar/nap* all come from anoxic samples and do not cluster together (Figure 2A).

**FIGURE 3:**
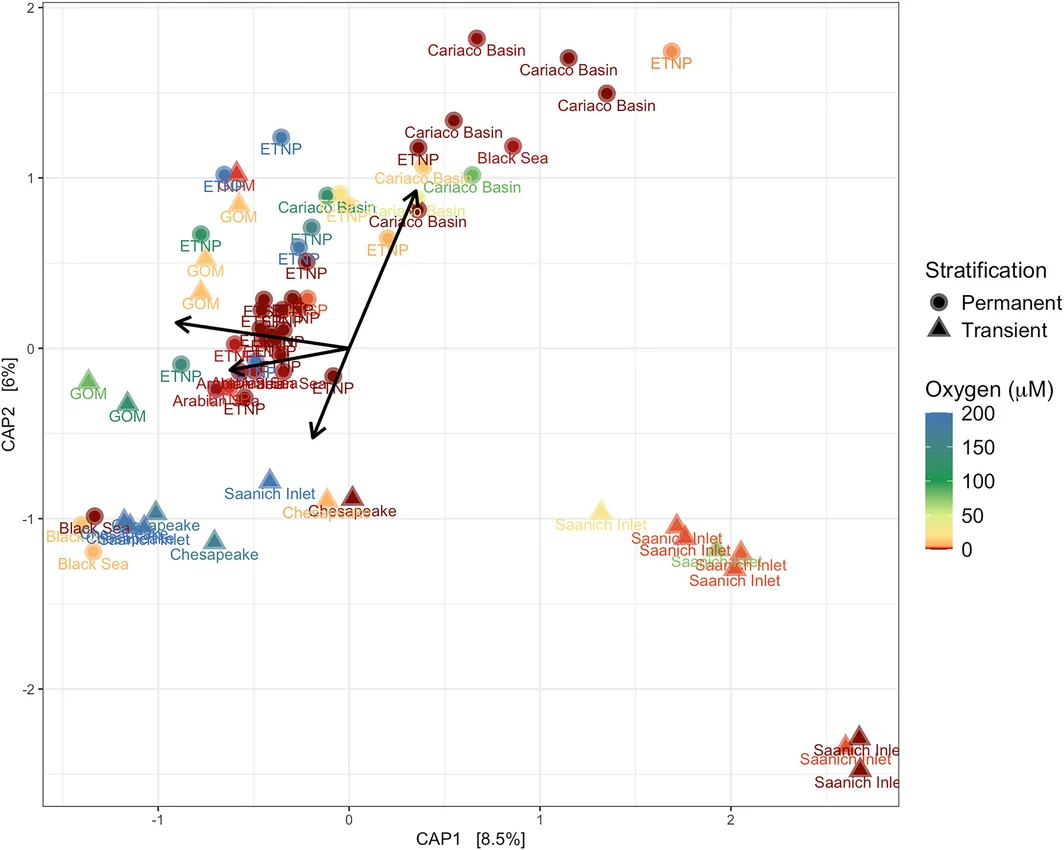
CAP plot based on Bray–Curtis dissimilarity of relative abundance of all denitrification modularity patterns in each sample. Transient sites are denoted with triangles and permanent sites are marked with circles. Oxygen is shown by the colour bar. Arrows show environmental variables (concentration of oxygen (O_2_), temperature (Temp), particulate organic carbon (POC) and salinity) significantly correlated with the ordination axes (*p* < 0.05).

The CAP analysis (Figure 3) further revealed that dissimilarity in relative abundance of modularity patterns between samples is driven by stratification type (permanent vs. transient) (*p*‐value = 0.001), location (*p*‐value = 0.001), temperature (*p*‐value = 0.004), oxygen concentration (*p*‐value = 0.009) and salinity (*p*‐value = 0.02), based on PERMANOVA results. Across all sites, however, geographic location appears to be the greatest indicator of modularity pattern dissimilarity (Figure 3). Most anoxic samples from OMZs, excluding the Cariaco Basin, group together and this grouping is driven by oxygen (Figure 3). Notably, the Saanich Inlet depths grouped separately, but the available environmental data were not able to explain the distance between Saanich Inlet and the rest of the sites.

**FIGURE 4:**
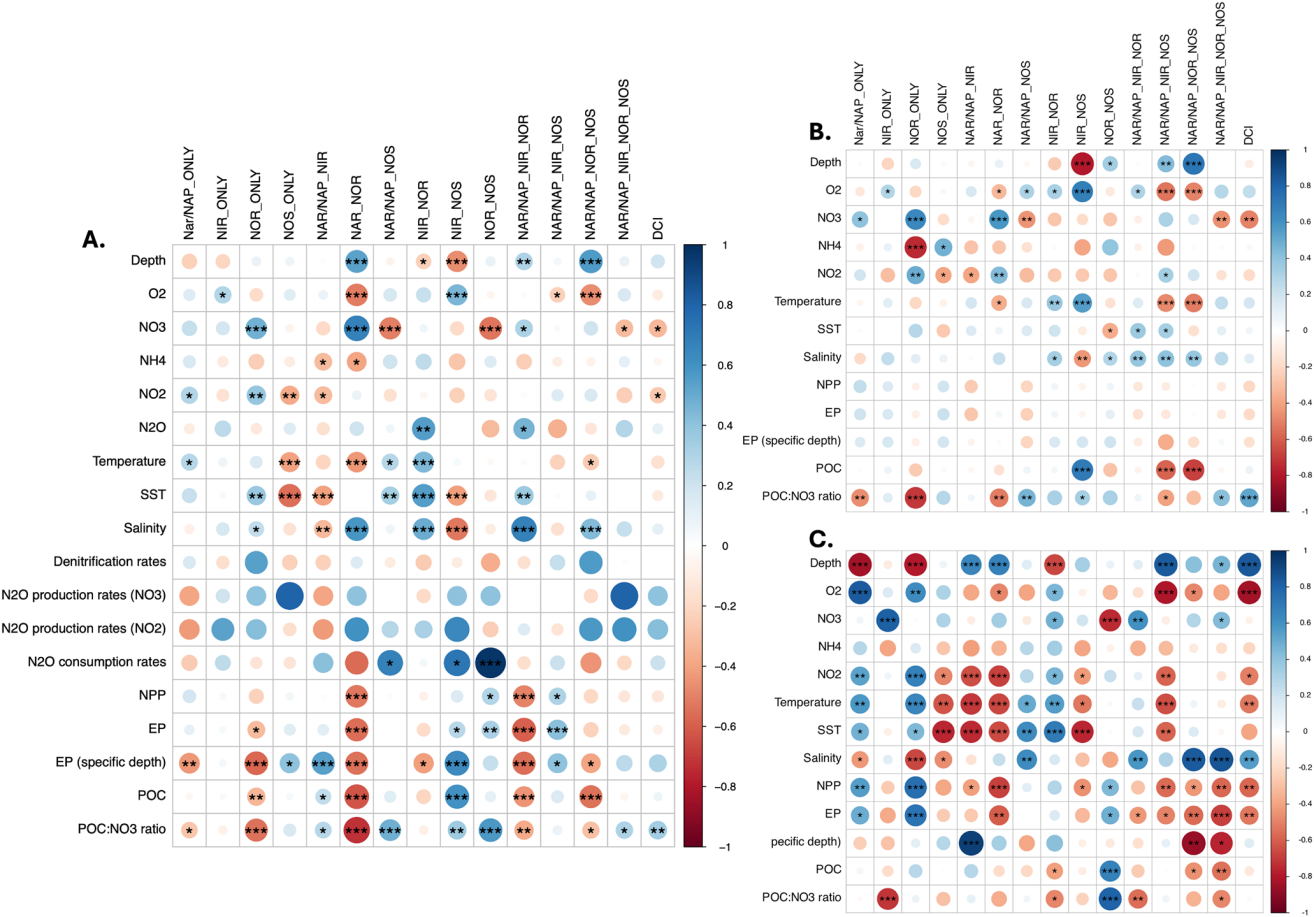
Spearman correlation coefficients (*ρ*) between relative abundance of modularity patterns and available environmental data for all samples (A), permanently stratified samples only (B) and transiently stratified samples only (C). Denitrification Completeness Index (DCI), sea surface temperature (SST), net primary production (NPP), export production (EP) and particulate organic carbon (POC) are all represented by their respective acronyms. The colour and size of the circle denote the strength of the correlation (magnitude of *ρ*) and stars represent statistical significance (* ≤ 0.05, ** ≤ 0.001, *** ≤ 0.0001). X and Y column order is manually set for clarity.

In both transient and permanent systems, *nar/nap* is the most abundant modularity pattern, with the notable exception of Saanich Inlet, where the most abundant modularity pattern is *nar_nir_nos*. Saanich Inlet also has high levels of POC as well as EP (Table S1). This supports previous theories from an ecological model that suggests more complete denitrification is favoured in higher OM environments (Sun et al. 2024). However, contradictory to that hypothesis, no complete denitrifier MAGs were recovered from the POC‐rich Chesapeake Bay. Chesapeake Bay is a relatively high OM environment, so by electron donor/acceptor availability and thermodynamic principles alone, one would expect the most complete denitrification at this site. In fact, in all transient systems, NPP, EP, EP at depth, POC, and POC:NO_3_^−^ are all significantly negatively correlated with complete denitrification (Figure 4C). Very low abundances of complete denitrifiers were mapped to the Chesapeake Bay metagenomes; however, the Bay had some of the highest abundance of *nar/nap* modularity patterns. *nar* and *nap* can have different energetic contributions, as some variations of NapA do not contribute to energy generation (González et al. 2006). *nar* and *nap* might have different responses to POC:NO_3_^−^, which might partially explain the lack of expected correlation between *nar/nap* and POC:NO_3_^−^. This explanation cannot be explored here because *nar/nap* were not separated in this analysis. None of the transient sites showed the thermodynamically predicted positive correlation between *nar/nap* and all proxies for OM supply, which we suggest could be related to tradeoffs imposed by the complexity of these environments.

Conversely, in the permanently anoxic samples POC:NO_3_^−^ is positively correlated with complete denitrification, matching with thermodynamic expectations. All transiently anoxic systems in this study are very dynamic and high flux environments, therefore, these negatively correlated patterns are not unusual as previously outlined ecological principles suggest these environments should favour shorter pathways for increased metabolic flexibility, nutrient uptake, stress responses, antibiotic production, etc. (Pfeiffer and Bonhoeffer 2004; Kreft et al. 2020; Roothans et al. 2025). More work is needed to untangle the thermodynamic and metabolic trade‐off controls; however, these results implicate both factors.

Complete denitrification (*nar/nap_nir_nor_nos*) is most abundant in the Cariaco Basin, which may be due to the high POC:NO_3_^−^ ratio and/or stability of hydrography in the basin (Figure 2). The lack of complete denitrifiers in the ETNP and ETSP is interesting because the rates of denitrification measured there (Thamdrup et al. 2006; De Brabandere et al. 2014; Babbin et al. 2020; Tracey et al. 2022) have long been implicitly attributed to complete denitrifiers. This suggests that most denitrification in these regions is accomplished by community cross‐feeding of many different denitrification modules working together.

Both permanent and transient systems have a relatively high abundance of pathways that terminate at the toxic intermediate NO (*nar/nap_nir*), as well as pathways that are missing *nor* (*nar/nap_nir_nos*) (Figure 2). Absence of *nor* and pathways ending in *nor* seem unlikely due to the known toxicity of NO. It is also possible, however, that this modularity type is environmentally relevant but missed in culture‐based work because dense cultures do not allow for the diffusion of NO out of the system and would force accumulation to a toxic level. Recent culture work with novel methods has allowed for the isolation of a denitrifier that is lacking Nor (Lycus et al. 2017). Furthermore, a recent study (Murali et al. 2021) unveiled large amounts of hidden diversity in the Nor enzyme resulting in four new clades (eNor, sNor, gNor and nNor) that are all involved in nitric oxide reduction. All of these novel clades, in addition to previously identified qNor, bNor and cNor clades, were included in this study. However, it is still possible that the samples in which these unique and unlikely modularity pathways/patterns lacking *nor* were found may contain other unidentified versions of nitric oxide reductases.

### 3.3 Modularity Patterns Correlate With Different Environmental Variables Between Transiently and Permanently Anoxic Systems

**FIGURE 5:**
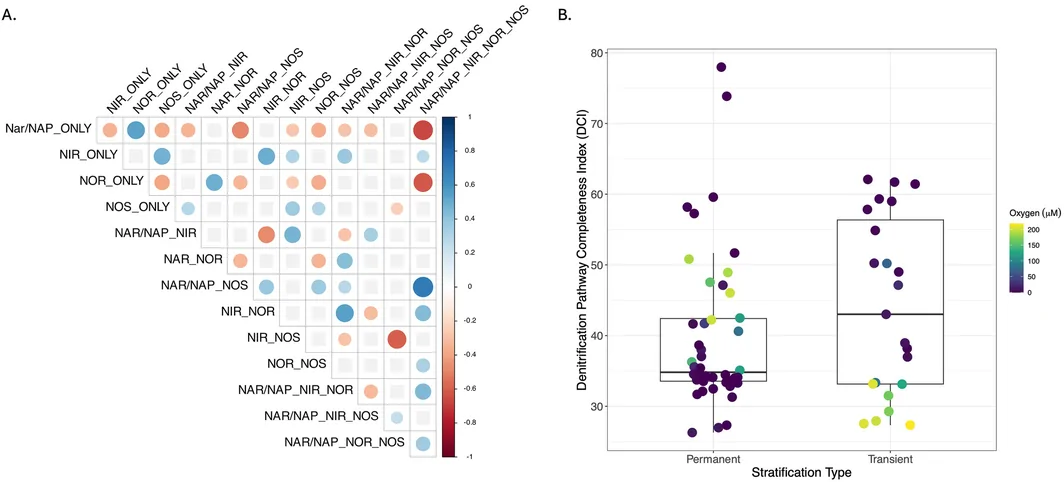
(A) Spearman correlation coefficients (*ρ*) between relative abundance of modularity patterns. The colour and size of the circle denote the strength of the correlation (magnitude of *ρ*), only significant correlations (*p* < 0.05) are shown with a coloured circle, grey squares are non‐significant. X and Y order is manually set for clarity. (B) DCI for permanent and transient sites coloured by in situ oxygen concentration; mid‐line represents the median.

To investigate potential shared niche spaces between modularity patterns, correlations between the relative abundance of each modularity pattern and a suite of environmental variables were calculated for all samples and separately for transient and permanently anoxic site types (Figure 4). Likewise, correlations between the relative abundances of all modularity patterns (i.e., co‐occurrence of the different patterns) were calculated for all samples (Figure 5A) and separately for transient and permanently anoxic environments (Figure S5). Overall, the environmental variables included here explain modularity type and abundance in transient systems better than in permanent systems, as there are more significant correlations in transient sites than permanent ones (Figure 4). This is not a result of sample size as there are fewer samples in the transient group than in the permanent group. The greater range and variability in time and space of environmental factors in transient sites than in permanent sites suggests that environmental factors drive patterns in denitrification modularity. This variability leads to more theoretical niche spaces for different modularity patterns in transient systems. However, similar numbers of modularity patterns are found in both transient and permanent systems (Figure 2).

Nevertheless, this multivariate control on modularity in transient systems may be why previous attempts to connect denitrification modularity patterns in metagenomic data to organic matter and DIN ratios on a global scale have shown non‐significant correlations (Pold et al. 2025). These findings challenge the assumption that OM availability alone favours long denitrification pathways and suggest an important role for additional factors such as metabolic tradeoffs and the community benefits of division of labour in a highly unstable environment that may select for short pathways despite the relatively high OM concentration.

POC:NO_3_^−^ ratio has been proposed as a driver of denitrification modularity patterns and is found here to be an important environmental correlate. When considering all samples, POC:NO_3_^−^ is negatively correlated with *nar/nap* and positively correlated with complete denitrifiers and the DCI (Figure 4A). This is consistent with thermodynamic expectations, measured rates in OMZs and modelling expectations (DeVries et al. 2013; Babbin et al. 2014; Tracey et al. 2022; Sun et al. 2024), but has not been observed in any other metagenomic modularity studies. Interestingly, this relationship is limited to permanently stratified anoxic systems whereas POC:NO_3_^−^ and proxies for OM supply in transiently stratified systems are all negatively correlated to complete denitrification (Figure 4C). This may be because the ecological models are parameterised at steady state, which is more similar to a permanently stratified OMZ than a high flux and dynamic environment like a transiently stratified estuary.

Carbon fluxes and concentrations were calculated from satellite data, which corresponded with the sampling date of the metagenome sample. That means that the in situ population could be the result of a previous transient event. In transiently anoxic systems, a pulse of OM would be consumed by aerobic respiration drawing down both OM and O_2_ concentrations, thus favouring denitrification in comparatively lower OM environments. The anoxia of permanently anoxic systems, such as OMZs, reflects a longer‐term balance between ventilation controlled by ocean circulation and decomposition of OM supplied by overlying primary production. Therefore, it is possible that the temporal variability of OM and its role in structuring microbial communities may not be captured in this dataset.

Measured N_2_O and N_2_ production rates and concentrations of intermediate DIN compounds often correlate with the modularity types that produce and consume the measured intermediates (Figure 4A). NO_2_^−^ concentrations in all samples and NO_3_^−^ concentrations in permanently anoxic samples are positively correlated with *nar/nap*, most likely because this process produces NO_2_^−^ and is favourable at high NO_3_^−^ concentration. NO_3_^−^ concentration was correlated with initiators of denitrification (defined as pathways containing *nir* but lacking *nos*) in a recent global overview of modular denitrification; however, a direct comparison is not possible because that study did not include *narG* or *napA* (Pold et al. 2025) and initiation of denitrification was assumed to be represented by *nir*. Interestingly, NO_2_^−^ concentrations are also negatively correlated with the denitrification completeness index (DCI) (Figure 4). This supports the hypothesis that an accumulation of NO_2_^−^ is due to greater abundance of partial compared to complete denitrifiers. N_2_O consumption rates are positively correlated with the *nor/nos, nar/nap_nos* and *nir_nos* modularities. Rate of N_2_O production tends to vary positively, but is not significantly correlated, with many different intermediate pathways, pointing towards a diverse assemblage of modularity patterns that result in the flux of N_2_O from denitrification. N_2_O concentration is positively correlated with pathways that produce N_2_O: *nir_nor* and *nar/nap_nir_nor*. These rates cannot be split by site type (permanent vs. transient) due to the low sample size of locations with available rate data. However, these correlations show that across all sample sites, modularity type can be predictive of measured rates and can provide insight into which functional gene patterns are responsible for their associated bulk measurements.

In transient systems, oxygen concentrations correlate positively with *nar/nap* and negatively with more complete pathways as well as pathways containing *nos* (Figure 4C). DCI is also negatively correlated with oxygen, and this trend is driven by the transient samples (Figure 4C, Figure 5B). These correlations are consistent with both oxygen sensitivity kinetics previously measured in Chesapeake Bay and with enzyme based studies, which demonstrate that N2OR is the most oxygen intolerant denitrification enzyme (Körner and Zumft 1989; Tang, Fortin, et al. 2025). In transient samples, DCI is driven by oxygen, with low O_2_ samples resulting in more complete denitrifiers and high O_2_ samples resulting in more truncated pathways (Figure 5B). However, these correlations with oxygen do not hold for permanently anoxic sites (which do include oxygenated surface samples), so it is possible that the oxygen sensitivities of these communities vary with the stability or permanence of anoxia (Figure 4B, Figure 5B).

### 3.4 Co‐Occurrence of Modularity Patterns

In addition to correlations with environmental factors, modularity types might also be expected to correlate with each other based on the potential for cross‐feeding or competition among microbes with different substrate requirements. Here we consider co‐occurrence of modularity types identified by their relationships to nitrate, nitrite and N_2_O distributions.

#### 3.4.1 NO_3_^−^ Availability

*Nar/nap* was strongly negatively correlated with the abundance of complete denitrifiers (Figure 5A). This finding is supported by both theoretical ecological principles and ecological models (Zhang et al. 2023; Sun et al. 2024). A potential driver of this correlation could be that complete denitrification is also negatively correlated with NO_3_^−^ availability, which is positively correlated with *nar/nap* (although only significantly so at the permanent sites) (Figure 4). These correlations support the hypothesis that higher electron acceptor (NO_3_^−^) availability selects for shorter pathways. Importantly, these negative correlations between complete denitrifiers and *nar/nap* and between complete denitrifiers and NO_3_^−^ availability are driven by permanently anoxic samples (Figure 4B). We hypothesise that the thermodynamic expectations hold true for permanent anoxia but not for transient anoxia because transiently anoxic systems have other environmental pressures (manifested by the stronger environmental correlations) and require trade‐offs to meet the needs for metabolic diversity and investment in survival tactics for unstable conditions.

#### 3.4.2 NO_2_^−^ Availability

**FIGURE 6:**
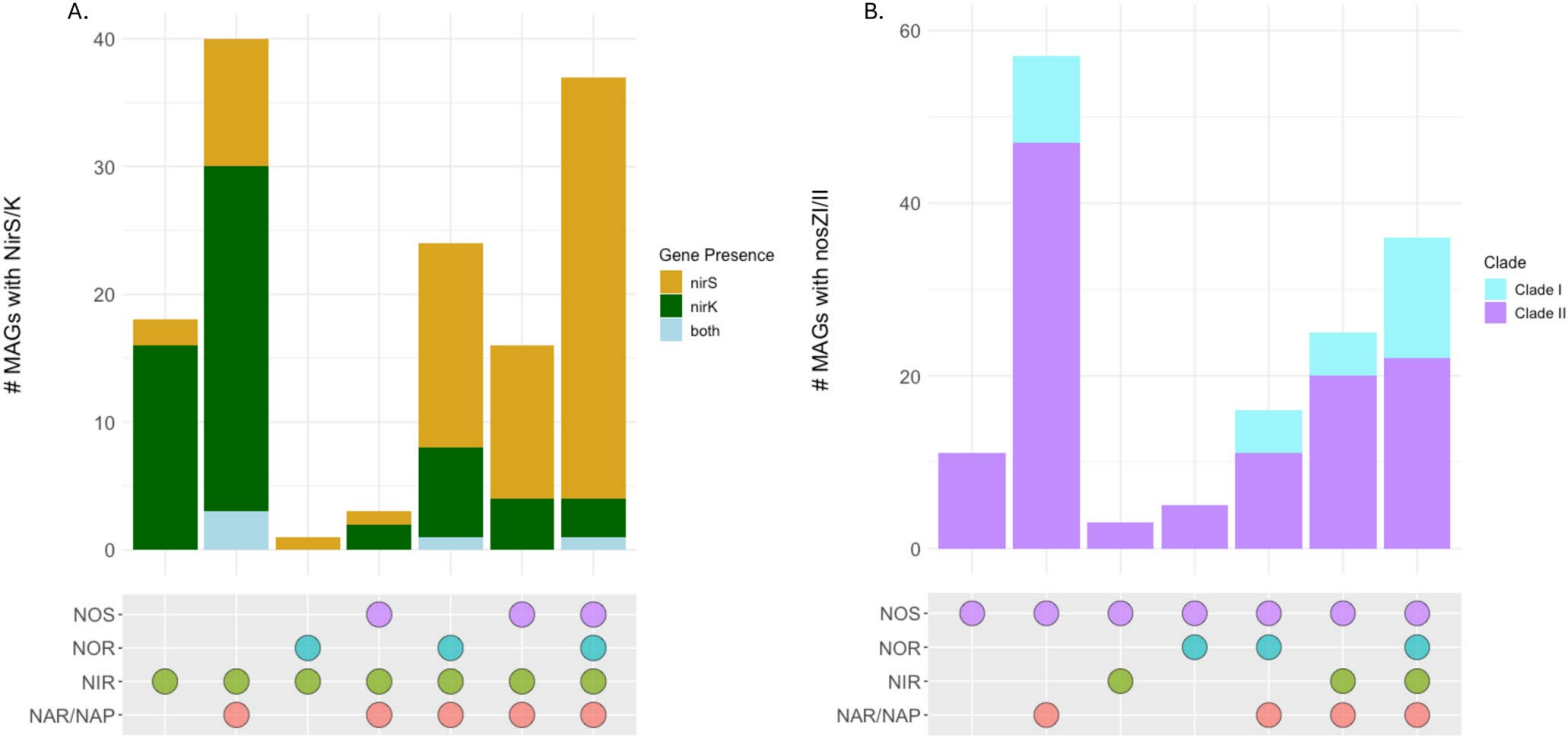
(A) Number of MAGs with *nirS* (yellow), *nirK* (green) or both (blue) and their associated modularity pattern. (B) Number of MAGs with Clade I and Clade II *nosZ* and their associated modularity pattern.

Modularity types starting with *nir* (including *nir* only) were not abundant in either transient or permanently anoxic environments and did not have significant correlations with ecologically meaningful environmental variables (e.g., were not correlated with nitrite concentration). The accumulation of NO_2_^−^ that is often associated with oxygen depletion is consistent with the much greater prevalence of *nar/nap* over *nir* modularities. *Nir* occurred most frequently together with *nar*/*nap* in various combinations and is positively correlated with complete denitrifiers in both environments. The major difference in *nir* modularity patterns was the almost complete absence of *nar/nap*_*nir*_*nos* in the permanently anoxic environments contrasted with the relatively high abundance of this pattern in the transient environments (Figure 2). *nirS* type denitrifiers have been previously shown to be more associated with complete denitrification than *nirK* type (Graf et al. 2014; Pold et al. 2024). The MAGs in this study support this finding as the vast majority of complete denitrifiers contain *nirS* and shorter pathways are dominated by *nirK* (Figure 6A). *NirK* containing MAGs were also more often removed during taxonomic filtering than *nirS* because they were more associated with known nitrifiers or anammox bacteria (Figure S2).

#### 3.4.3 N_2_O

The addition of *nosZ* in a modularity pattern is of great environmental importance because it encodes the enzyme responsible for reducing N_2_O to N_2_, thereby mitigating emissions of this potent greenhouse gas. In permanently anoxic systems, the percent abundance of all modularity patterns containing *nos* are among the least common (Figure 2). *Nos* is on average 1.16% abundance of the total denitrifier MAG abundance, and all modularity types with *nos* make up 20.96% of the total denitrifier MAG abundance. The relative rarity of *nos* modularities in OMZs is consistent with the fact that these sites are large sources of N_2_O, accounting for up to 50% of global ocean N_2_O flux (Codispoti 2010; Ji, Buitenhuis, et al. 2018) while transient systems like Chesapeake Bay are weaker and more variable sources of N_2_O, depending on redox conditions (Elkins et al. 1978; Tang, Tracey, et al. 2022). Notably, the POC:NO_3_^−^ ratio was positively correlated with almost all *nos* containing pathways in permanent systems (Figure 4). *Nos* MAGs are negatively correlated with *nar/nap* (Figure 5) and positively correlated with EP at depth (Figure 4). This is contradictory to the thermodynamic favourability of N_2_O reduction, which requires an excess of N per mole of C to effectively generate a proton motive force (*pmf*). However, pure thermodynamic favourability does not account for enzyme configuration and may be an overestimation of the bioenergetic potential of N_2_O reduction. There is currently no evidence directly linking *nosZ* to *pmf* generation potential (Simon and Klotz 2013); however, there is evidence of pure cultures able to respire on N_2_O alone (Conthe et al. 2018; Suenaga et al. 2019). The results reported here suggest that *nosZ* is favoured by a high POC:NO_3_^−^ ratio and is very rare in anoxic water columns. Previous genomic and rate data found that *nosZ* and N_2_O consumption rates are the most particle‐associated step of the denitrification pathway (Ganesh et al. 2015). However, large anoxic marine particles (a significant source of OM) are rare. Further work is necessary to understand the environmental control on the inclusion or exclusion of *nosZ* in the denitrification respiratory pathway.

*Nos* is one of the two denitrification genes, along with *nar*, that is implicated in bet‐hedging and may serve an ecological role in preventing entrapment in anoxia (Lycus et al. 2018). This survival mechanism would provide an explanation for why denitrification genes may be retained in fully oxygenated environments that have the potential to become anoxic. The relative abundance of the *nar/nap_nos* modularity is negatively correlated with oxygen availability in transient systems but not permanent systems (Figure 4B,C). This finding suggests that bet‐hedging may be a unique microbial solution for transiently anoxic environments and further supports that denitrification modularity differs greatly based on the stability of anoxia.

To further investigate these *nos* containing modularity patterns, the gene was classified as clade I, II or III, although clade III was not included separately in downstream analysis due to its high sequence similarity to either clade I or II (Figure S4). The vast majority of *nos* containing modularity types have clade II *nosZ* (Figure 6B). The proportion of CladeI:II increases as the modularity pathway becomes more complete. However, in all of these marine sites, even complete denitrifiers are majority clade II. This is in contrast to previous modularity studies, which found the majority of complete denitrifiers in globally distributed environments harbour clade I (Graf et al. 2014; Pold et al. 2025; Roothans et al. 2025). It may be that the much bigger terrestrial sequence database obscured the opposite pattern observed here in marine systems. Based on these results, *nosZ* clade assignment is not a good proxy for the completeness of denitrification.

## 4 Conclusions

Denitrifiers in both transient and permanently stratified anoxic systems are dominated by short, truncated pathways. However, not all short pathways are created equal. The great over‐representation of the first step of denitrification may be driven by the low OM supply and POC:NO_3_^−^ ratio of the environments studied, which represent most of the open ocean. This over‐representation also highlights the thermodynamic favourability of nitrate reductase (encoded by *nar*) due to its unique ability to directly pump protons. While this first step is often excluded from denitrification studies because it doesn't directly result in the loss of fixed N, it is ubiquitous in denitrification communities and provides the substrate that does lead to complete denitrification (NO_2_^−^) and growth of denitrifiers. It is still unknown why *nir* modularity patterns are so rare while microorganisms with the capability of producing NO_2_^−^ and no way to reduce it are over‐represented; more work is needed to understand this paradox.

*Nos* containing modularity types are noticeably scarce across all samples and are positively correlated with OM supply proxies and POC:NO_3_^−^ ratio, which provides further support for the particle‐attached lifestyle proposed for N_2_O respiring microorganisms. More work is needed to understand when it is environmentally favourable to reduce N_2_O in order to constrain when denitrification communities act as a source or sink of this potent greenhouse gas.

The permanence of stratification, oxygen availability, temperature, salinity and biogeographic location all help to explain the variance in modularity pattern abundance among sites. However, the strongest variable found here is the permanence of stratification, along with biogeographic location. More importantly, a key difference between the two site types is their differential response in terms of modularity distributions to ecological controls.

The relationship between estimated POC:NO_3_^−^ ratio and modularity is consistent with modelled and thermodynamic expectations (positively correlated to complete denitrifiers and negatively correlated to *nar/nap*). However, this finding is only significant in permanently stratified systems. We hypothesise that this may be because transiently anoxic systems create other ecological pressures (i.e., metabolic trade‐offs), which is consistent with the higher number of significant environmental correlations for the transient sites. Another explanation is that permanently anoxic sites may better match findings from models that are parametrised at steady state, as compared to high‐flux transiently anoxic environments. This finding highlights the importance of including all types of anoxia in future modularity studies, as our current understanding of the controls on modularity appear to apply only to OMZ‐like systems. This may also provide an explanation for why it can be difficult to pinpoint environmental controls on denitrification modularity across global datasets, because the stability of an environment plays a significant role in determining which denitrification gene combinations are favourable.

## Author Contributions

**Samantha G. Fortin:** methodology, writing – review and editing. **Bess B. Ward:** conceptualization, writing – original draft, funding acquisition, supervision, resources, methodology, project administration. **J. Catherine Hexter:** writing – original draft, conceptualization, data curation, formal analysis, methodology, investigation, visualization. **Weiyi Tang:** formal analysis, writing – review and editing. **Amal Jayakumar:** investigation, writing – review and editing.

## Funding

This work was supported by the National Science Foundation (OCE‐2342493) and Department of Geosciences, Princeton University, Myhrvold‐Havranek Graduate Fellowship.

## Conflicts of Interest

The authors declare no conflicts of interest.

## Supporting information


**Figure S1:** Station map for all metagenomic samples used in this study representing a depth profile of comparable features in both transiently and permanently anoxic marine systems. Average 2018 oxygen concentrations at 250 meters depth from World Ocean Atlas (WOA) displayed by underlying color (Garcia et al. 2024).

**Figure S2:** Maximum Likelihood (ML) phylogenetic tree of translated NirS amino acid sequences annotated from MAGs and reference NirS sequences (grey) (Pold et al. 2024). Color strips denote annotation (presence is blue and absence is white) of the remaining denitrification genes in the associated MAG. This information is not provided for the references sequences nor for MAGs that were removed due to phylogeny since these sequences were not included in any downstream analysis (see methods).

**Figure S3:** Maximum Likelihood (ML) phylogenetic tree of translated NirK amino acid sequences annotated from MAGs and reference NirK sequences (grey) (Pold et al. 2024). Color strips denote annotation (presence is blue and absence is white) of the remaining denitrification genes in the associated MAG. This information is not provided for the references sequences nor for MAGs that were removed due to phylogeny since these sequences were not included in any downstream analysis (see methods).

**Figure S4:** Maximum Likelihood (ML) phylogenetic tree of translated N2OR amino acid sequences annotated from MAGs. First color bar denotes clade assignment (Clade I (blue) and Clade II (purple)) confirmed via BLASTp, CDHIT clustering with known references, and tree morphology. The outer color bar denotes genes who sequences were identified as Clade III via the conserved motif described in (He et al. 2025).

**Figure S5:** Spearman correlation coefficients (*rho*) between relative abundance of modularity patterns for both (A) transiently and (B) permanently anoxic samples. The color and size of the circle denote the strength of the correlation (magnitude of *rho*) and non‐significant correlations are shown by grey squares. X and Y order is set by hierarchical clustering.

**Table S1:** Metagenome sample metadata including all environmental parameters, sample citations, cruise, date, latitude, longitude and SRR number.
**Table S2:** MAG metadata for all MAGs including non‐denitrifiers including contamination (%) and completion (%).
**Table S3:** GCA numbers for NirS and NirK reference sequences used to validate phylogenetic trees.
**Table S4:** *nosZ* clade assignments.
**Table S5:** Brief descriptions and original citations for methods used to collect all biogeochemical data used in this study.
**Table S6:** MAG metadata for MAGs included in study post QC including taxonomy, denitrification gene presence, modularity patterns and denitrification relative abundance (%).
**Table S7:** Shannon diversity, richness and Simpson diversity for all modularity patterns across all sample sites.

## Data Availability

All newly created metagenomically assembled genomes for this study have been deposited in the NCBI GenBank repository under accession number PRJNA1472692. All newly sequenced metagenomes have been deposited under accession number PRJNA1151642. Accession numbers for previously published metagenomic datasets used in this study are listed in Table S1.
